# “Nothing Can Stop Me!” Perceived Risk and Travel Intention Amid the COVID-19 Pandemic: A Comparative Study of Wuhan and Sapporo

**DOI:** 10.1007/978-3-030-65785-7_47

**Published:** 2020-11-28

**Authors:** Si Ru Li, Naoya Ito

**Affiliations:** 1 Department for Informatics, Technical University of Munich, Garching bei München, Bayern Germany; 2 Smart Tourism Education Platform (STEP) College of Hotel and Tourism Management, Kyung Hee University, Seoul, Korea (Republic of); 3 Department of Tourism and Service Management, MODUL University Vienna, Vienna, Wien Austria; 4 Graduate School of International Media, Communication, and Tourism Studies, Hokkaido University, Sapporo, Japan; 5 Research Faculty of Media and Communication, Hokkaido University, Sapporo, Japan

**Keywords:** COVID-19, ELM, Perceived risk, Travel intention

## Abstract

The global tourism industry has been devastated by the COVID-19 pandemic due to strict travel restrictions imposed by most countries. In order to achieve a swift post-pandemic recovery, it is important to understand what psychological obstacles people would face when making travel decisions. Building upon the dual-route theory of information processing, this study examined and compared how the perceived risk of COVID-19 would affect people’s travel intentions in the Japanese city of Sapporo and the Chinese city of Wuhan through two rounds of data collection. While both cities were hit hard by the COVID-19 pandemic at an early stage, the cumulative numbers of confirmed human cases and the levels of intervention adopted were largely different. Results from the present study showed that risk perception of COVID-19 had a negative effect on people’s travel intentions in Sapporo. However, no significant effect of COVID-19 perception could be observed in post-lockdown Wuhan. Meanwhile, although the dual-route structure of information processing was obtained in Sapporo and post-lockdown Wuhan, neither routes seemed to predict the perceived risk of COVID-19 in Wuhan when lockdown restrictions were still in place. Several theoretical and practical implications concerning the results are discussed in this study.

## Introduction

The year 2020 could have been another promising year for the global tourism industry because of the Tokyo Summer Olympics and other major cultural events [1]. However, the unprecedented outbreak of COVID-19 reminded people how susceptible tourism is to various risks and threats [2]. Prior to the COVID-19 pandemic, both China and Japan saw increasing inbound tourism demands. In 2018, Chinese visitors ranked first on tourism expenditure in Japan with 1.545 trillion yen [3]. Meanwhile, Japan was also said to be one of the leading source countries of China’s inbound tourists [4]. Nevertheless, inbound tourism has been put on halt under the current pandemic. Even if the disease can be contained through global joint efforts, the perception of health hazard and lack of safety may persist and deter people from travelling in the near future [5].

People’s travel decisions and behaviours are largely influenced by their risk perceptions, which are formed from information that does not necessarily reflect the reality [2]. While people’s travel intention can be enhanced by positive online reviews [6], it can also be adversely affected by negative opinions and misperceptions especially when a destination is linked to a contagious disease [7]. If people perceive high health risk towards a destination, it is less likely for them to visit there out of safety concerns [5]. Since the virus was first identified in the Chinese city of Wuhan, COVID-19 was sometimes referred to as “Wuhan virus” or ‘Chinese virus” on social media in the early stage of the outbreak [8]. In order to regain people’s confidence in COVID-19 stricken destinations, local authorities and tourism practitioners should understand how and to what extent people are affected by information related to the current pandemic. Thus, the first goal of the present study is to ascertain how people process COVID-19 information to form their perceived risk of the disease and whether such perception would influence their intentions to visit Wuhan or China.

Meanwhile, people from different countries may perceive risks differently due to geographical and cultural variations [9]. This study compares the perceived risks of COVID-19 in China and Japan by conducting quantitative surveys in Wuhan and Sapporo in the early stage of the outbreak. Wuhan, known as the former epicenter of COVID-19, was placed under lockdown for 76 days since January 23, 2020 [10]. On the other hand, Sapporo was among the first Japanese cities to be affected by the disease as early as mid-February [11]. Unlike Wuhan however, Sapporo never went into a compulsory lockdown besides being placed under a state of emergency [12]. Given that the total numbers of infections and the levels of interventions in controlling the disease were different in these two cities, evident regional difference in COVID-19 perception would be expected. In addition, the present study recognizes that different stages of COVID-19 control could have varying influence on perceived risk and travel intention. In order to figure out whether the effect of COVID-19 risk perception would persist after the pandemic, additional data are necessary from regions that have successfully combated the disease. Therefore, the second objective of this study is to investigate any possible changes in people’s travel intentions during and after the pandemic by obtaining data from post-lockdown Wuhan.

## Theoretical Background

### Perceived Risk and Travel Intention

Intentions are indications of how much people are willing to engage in a behaviour [13]. In the filed of tourism, an individual’s subjective norm, perceived behavioural control, and past travel behaviour are considered to be important predictors of travel intention [14]. Yet, within the current pandemic situation, these predictors became largely uncontrollable due to border closures and strict travel restrictions. In the absence of personal experience, people’s perceptions of risk and safety come into play when making travel decisions [15]. Prior literature has identified five major risk factors related to tourism: (1) war and political instability, (2) health concerns, (3) crime, (4) terrorism, and (5) natural disasters [16]. Within those, health risk perception was found to come second after crime-induced risk when planning a trip to developing countries [17]. As COVID-19 has developed into a global pandemic, people are placed under the risk of infection not only in the developing world but also in high-income countries. This study defines perceived risk as the subjective belief that an individual will experience uncertain negative outcomes because of COVID-19.

#### H1.

Risk perception of COVID-19 has a negative effect on travel intention.

### Argument Quality and Source Credibility

While the impact of risk perception on travel has been an active research topic [9, 18], there is still scant knowledge regarding how people process information to construct their risk perceptions. Among all research approaches to investigate people’s information processing behaviours, the dual-route theory of information processing is deemed appropriate for predicting risk judgment [19, 20].

The elaboration likelihood model (ELM) is a dual-route theory developed by Petty and Cacioppo [21], which suggests two different modes of information processing. Based on their theory, the first mode of processing, known as the central route, results from an individual’s thoughtful evaluation of a message. In contrast, information processing can also occur through the peripheral route, which is induced by simple cues in the message without thorough scrutiny of the content itself. The central processing route would be adopted by people with higher elaboration likelihood and result in enduring attitude change, whereas the peripheral processing route would be favoured by people with lower elaboration likelihood [21]. Prior literature has identified argument quality and source credibility as two distinct variables that represent the central and peripheral routes of processing, respectively [22].

Endeavour has been made to associate the dual-route processing theory with risk perception. For instance, Trumbo [19] evaluated the heuristic-systematic model (HSM), a dual-process model similar to the ELM, for its ability to predict people’s risk judgments on a suspected cancer cluster. He found that heuristic processing (peripheral route) was linked to lower risk judgment while systematic processing (central route) was associated with greater risk judgment. In a study that measures people’s perceptions of the Fukushima nuclear accidents, systematic processing was found to result higher perceived risk whereas heuristic thinking had no significant influence [20]. Since heuristic processing involves less cognitive evaluation of risk information [23], people who do not scrutinize COVID-19 information carefully may underestimate the risk of the disease due to optimistic bias [24].

#### H2.

Argument quality of COVID-19 information intensifies risk perception.

#### H3.

Source credibility of COVID-19 information mitigates risk perception.

### Self-efficacy

**Fig. 1.**
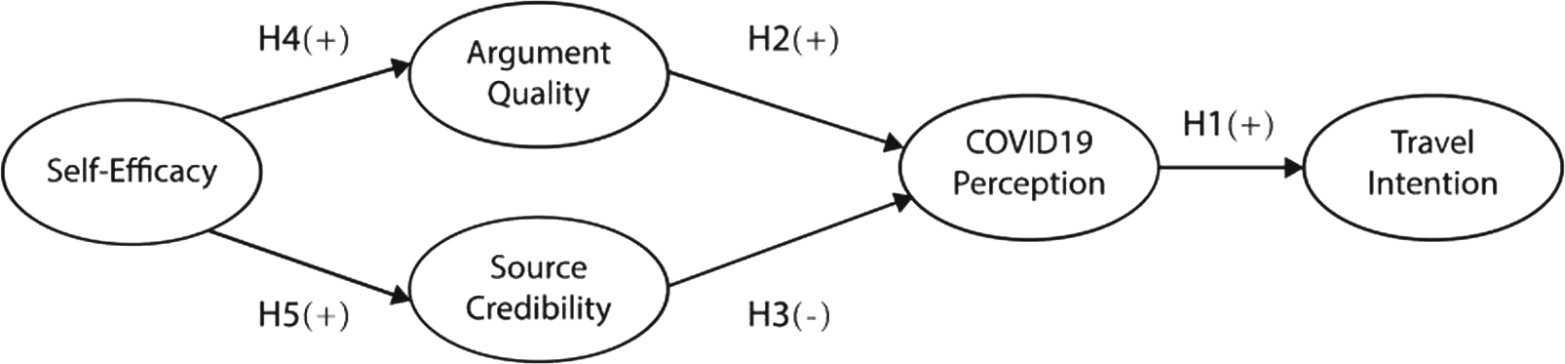
Research model (compiled by authors)

As noted previously, different modes of information processing are selected based on an individual’s elaboration likelihood, which is determined by his or her’s motivation and ability to evaluate the information [21]. However, ability is not a fixed attribute of a person. Rather, it resides in people’s self-beliefs of their capabilities to perform an activity [25]. Moreover, most motivation is cognitively generated to guide one’s actions. During academic learning, motivation is enhanced when students believe that they are performing well at school [26]. Hence, an individual’s ability and motivation to perform a certain action might be explained by his or her sense of self-efficacy. The present study defines self-efficacy as people’s perceived ability to collect and comprehend COVID-19 information, which predicts the effects of the central and peripheral routes (Fig. 1).

#### H4.

Self-efficacy positively affects argument quality of COVID-19 information.

#### H5.

Self-efficacy positively affects source credibility of COVID-19 information.

## Methodology

### Measurement

Residents of Wuhan and Sapporo were surveyed about any COVID-19 information they received from the authorities, mass media, websites, social media, or face-to-face interactions. All constructs in the survey were adapted from the literature and measured with a 7-point Likert scale, ranging from 1 (strongly disagree) to 7 (strongly agree). In this study, argument quality (AQ) reflects accuracy, timeliness, and completeness of COVID-19 information that respondents received through different channels [22]. Items for argument quality were modified from existing studies on online reviews, which use the dual-route model to predict consumers’ information adoptions and behavioural intentions [22, 27]. Items for source credibility (SC) were adapted from the work of Yoo et al. [28], in which source credibility is used as a peripheral cue to capture respondents’ perceived credibility of a tourism website in terms of professionalism, trustworthiness, and reliability. Based on Li, Guo, and Ito [29], self-efficacy (SE) can be used as a predictor of people’s information seeking behaviours in a risk communication setting. Thus, self-efficacy was operationalized in this study as a construct to measure respondents’ beliefs in their capacities to collect and understand COVID-19 information. Items for perceived risk of COVID-19 (PC) and travel intention (TI) were both adapted from the study of Lee et al. [18], where the influence of H1N1 influenza on travel intention is discussed.

Travel intention assessed for the Sapporo sample referred specifically to Wuhan and China because of their strong associations with COVID-19 in the opinions of some social media users [8]. It is worth finding out if risk perception induced by COVID-19 information would elicit negative views towards visiting Wuhan and China in the future. Respondents in Wuhan were not asked about travel intentions since the city was still in lockdown during the first round of data collection.

Additional data were obtained from post-lockdown Wuhan four months later to ascertain whether the risk perception of COVID-19 would still deter people from travelling in the post-pandemic era. People were surveyed about their intentions to visit any destination given that travel restrictions had been lifted in China at that time. Another data collection was not conducted in Sapporo due to the fact that the disease was far from being successfully contained in the city. In August 2020, Sapporo recorded 235 newly confirmed human cases [11], while Wuhan reported only 4 newly imported cases in the same month [30].

### Data Collection

#### Sapporo Sample.

An online survey was conducted in Sapporo through a Japanese research panel supplier in early April 2020. Survey items were first translated into Japanese and then translated back into English by two different researchers with vast experience of English-Japanese translation. There were no significant discrepancies between the original and the back-translated version. Overall, 542 valid responses were collected. Basic demographic information is listed in Table 1.

**Table 1.** Demographics of respondents (compiled by authors)

Characteristics		Sapporo (*N *= 542) April 2020		Wuhan (*N *= 516) March 2020		Wuhan (*N *= 400) August 2020	
		Frequency	%	Frequency	%	Frequency	%
Gender	Male	270	49.8	260	50.4	141	35.3
	Female	272	50.2	256	49.6	259	64.8
Age	Below 20	–	–	12	2.3	50	12.5
	20–29	105	19.4	141	27.3	225	56.3
	30–39	113	20.8	147	28.5	124	31.0
	40–49	104	19.2	153	29.7	1	.3
	50 and above	220	40.6	63	12.2	–	–
Education	High school or below	176	32.5	74	14.3	20	5.0
	College	150	27.7	108	20.9	44	11.0
	Bachelor’s degree	195	36.0	278	53.9	270	67.5
	Master’s degree/PhD	21	3.9	56	10.9	66	16.5

#### Wuhan Sample (First Collection).

Survey questions were distributed through one of the biggest online survey platforms in China in late March 2020. All survey questions were first translated into Chinese and then translated back into English by a group of researchers with sufficient knowledge of both languages. After excluding incomplete responses, a total of 516 valid responses were obtained from the city of Wuhan.

#### Wuhan Sample (Second Collection).

Another survey was conducted in post-lockdown Wuhan in mid-August 2020. Most parts of the survey remained identical from the previous version except for items measuring travel intention. A back-translation procedure was performed once again to ensure consistency in terminology. Overall, 400 valid responses were obtained from the city.

## Data Analysis

### Exploratory Factor Analysis

#### Sapporo Sample.

An exploratory factor analysis (EFA) was performed with maximum likelihood extraction method in SPSS 25.0. The result of the Bartlett’s test of sphericity confirmed that all variables were related and suitable for structure detection (*p* < .001). The minimum requirement of the Kaiser-Meyer-Olkin Measure (KMO) was met with a value of 0.863, which indicated that the sample was sufficient for further analysis [31]. Five significant factors were identified, which explained 79.601% of the total data variance. Following a promax rotation, one item from argument quality (AQ5) and one item from risk perception (PC4) were eliminated due to cross-loading problem. Cronbach’s alpha coefficients were calculated to ensure internal consistencies. All values were above 0.7, suggesting an overall high reliability [32].

#### Wuhan Sample (First Collection).

According to the EFA, both the Bartlett’s test of sphericity (*p* < .001) and the Kaiser-Meyer-Olkin Measure (KMO = .847) had satisfied the minimum requirements [31]. Overall, four significant factors were extracted, explaining 69.614% of the total data variance. After a promax rotation, one item from argument quality (AQ2), one item from source credibility (SC5), and one item from risk perception (PC4) were removed due to cross-loading issues. All Cronbach’s alpha coefficients were above 0.7, with the lowest found in COVID-19 perception (Cronbach’s α = .750).

#### Wuhan Sample (Second Collection).

Exploratory factor analysis was conducted to evaluate sampling sufficiency. Results from both the Bartlett’s test of sphericity (*p* < .001) and the Kaiser-Meyer-Olkin Measure (KMO = .869) confirmed that the sample was suitable for structure detection [31]. Five significant factors were extracted by using the maximum likelihood method. Cronbach’s alpha coefficients of all constructs were above 0.6. Since some of the Cronbach’s alpha values barely reached the acceptable level, results should be interpreted with caution (Table 2).

**Table 2.** Exploratory factor analysis results (compiled by authors)

Items	Sapporo (Apr 2020)		Wuhan (Mar 2020)		Wuhan (Aug 2020)	
	Loading	Cronbach’s α	Loading	Cronbach’s α	Loading	Cronbach’s α
**Argument quality**		.802		.815		.721
AQ1. Information I received about COVID-19 is accurate	.592		.524		.542	
AQ2. Information I received about COVID-19 is relevant to my need	.340		–		.296	
AQ3. Information I received about COVID-19 is comprehensive	.569		.843		.761	
AQ4. Information I received about COVID-19 is up-to-date	.692		.608		.533	
AQ5. Arguments of the information I received about COVID-19 are convincing	–		.734		.645	
**Source credibility**		.944		.881		.840
SC1. People providing information about COVID-19 are knowledgeable on the topic	.715		.627		.502	
SC2. People providing information about COVID-19 have experience dealing with infectious diseases	.783		.693		.503	
SC3. People providing information about COVID-19 are trustworthy	1.025		.914		.876	
SC4. People providing information about COVID-19 are reliable	.964		.940		.797	
SC5. People providing information about COVID-19 are professional	.832		–		.752	
**Self-efficacy**		.902		.785		.719
SE1. I have confidence in my ability to search on COVID-19 related information	.755		.788		.471	
SE2. I have confidence in my ability to understand COVID-19 related information	.900		.764		.758	
SE3. I have confidence in my ability to evaluate the credibility of COVID-19 related information	.962		.582		.346	
**Risk perception**		.846		.750		.623
PC1. COVID-19 is a frightening disease	.891		.848		.742	
PC2. I am afraid of contracting COVID19	.837		.709		.558	
PC3. Compared to SARS and Avian Influenza, COVID-19 is more dangerous	.735		.597		.499	
PC4. It is dangerous to travel because of COVID-19	–		–		.407	
**Travel intention (Sapporo)**		.909				
TIS1. I intend to visit Wuhan in the next 12 months	.841		–		–	
TIS2. I intend to visit China in the next 12 months	1.001		–		–	
**Travel intention (Wuhan)**						.892
TIW1. I intend to travel in the near future	–		–		.845	
TIW2. I am planning to travel in the near future	–		–		.852	
TIW3. I will make an effort to travel in the near future	–		–		.806	
TIW4. I will certainly invest time and money to travel in the near future	–		–		.795	

*Note.* Extraction Method: Maximum Likelihood. Rotation Method: Promax with Kaiser Normalization.

### Hypothesis Testing

All hypotheses were tested with regression analyses using SPSS 25.0. Regression analysis was chosen instead of the widely used structural equation modeling (SEM) approach because of the exploratory nature of the present study. While all measurement items were adapted from the extant literature, this study might be one of the first attempts to examine the dual-route theory in an ongoing pandemic. Also, since some studies have questioned the validity of the dual-route information model [33], SEM might not be the best choice in this study as it requires a sound theoretical base [34]. Meanwhile, the present study was not intended to substitute path analysis or SEM with linear regression analysis. Instead of dealing with the causal relationship structure between each variable, all regression analyses conducted in this study merely served the propose of capturing the direct effects from the included independent variables to the dependent variable [35].

#### Sapporo Sample.

A series of regression analyses was conducted to examine the previously stated hypotheses. Supporting H1, people’s perceptions of COVID-19 had a significant negative effect on their intention to visit Wuhan and China (*F* = 23.810, *p* < .001). Argument quality and source credibility significantly predicted COVID-19 perception (*F* = 47.081, *p* < .001). In addition, a positive influence was observed from AQ (β = .458, *p* < .001), whereas source credibility exerted a negative impact on COVID-19 perception (β = −.115, *p* < .05). Thus, H2 and H3 were validated. Supporting H4, self-efficacy positively influenced people’s perceived argument quality of COVID-19 information (*F* = 54.824, *p* < .001). H5 was also proven to be true as self-efficacy showed a positive influence on perceived source credibility of COVID-19 information (*F* = 47.812, *p* < .001) (Table 3).

**Table 3.** Results of regression analyses (compiled by authors)

Hypotheses	Sapporo (Apr 2020)		Wuhan (Mar 2020)		Wuhan (Aug 2020)	
	Standardized β	Result	Standardized β	Result	Standardized β	Result
H1. Perceived Risk → Travel Intention	−.205***	Supported	–	–	.006	Rejected
H2. Argument Quality → Perceived Risk	.458***	Supported	.100	Rejected	.193**	Supported
H3. Source Credibility → Perceived Risk	−.115*	Supported	−.081	Rejected	.136*	Partly Supported
H4. Self-Efficacy → Argument Quality	.304***	Supported	.619***	Supported	.538***	Supported
H5. Self-Efficacy → Source Credibility	.285***	Supported	.419***	Supported	.454***	Supported

*Note.* *p < .05. **p < .01. ***p < .001.

#### Wuhan Sample (First Collection).

Since travel intention was not measured in the Wuhan survey, H1 was excluded from the analysis. Hypothesis 2 and 3 were tested by conducting a multiple regression analysis. Contrary to the widely held assumption, neither of the processing routes had any significant effect on the risk perception of COVID-19 (*F* = 2.162, *p* = .116). Hence, H2 and H3 were both rejected. Linear regression analyses were performed to investigate whether self-efficacy could predict the two information processing routes. Supporting H4, self-efficacy positively predicted argument quality (*F* = 319.857, *p* < .001). Source credibility was also predicted by self-efficacy (*F* = 109.571, *p* < .001), which lent support to H5.

#### Wuhan Sample (Second Collection).

Regression analyses were performed in SPSS 25.0 to examine the previously stated hypotheses. Contrary to H1, COVID-19 perception in post-lockdown Wuhan did not affect their intentions to travel (*F* = .014, *p* = .907). Both argument quality and source credibility significantly predicted COVID-19 perception (*F* = 18.466, *p* < .001). However, the effect of source credibility on COVID-19 perception was also positive (β = .136, *p* < .05). Therefore, H2 was supported while H3 was partly supported. Self-efficacy positively influenced people’s perceived argument quality of COVID-19 information (*F* = 205.223, *p* < .001), supporting H4. H5 was also validated as self-efficacy showed a positive influence on the perceived source credibility of COVID-19 information (*F* = 103.564, *p* < .001).

## Discussion

### Temporary Effect of COVID-19 Perception

According to the Sapporo data, people’s perceived risk of COVID-19 indeed negatively influenced their intentions to travel during the early stage of the pandemic. Similar result can be found from a study on the Fukushima nuclear accident, where revisit intention was also directly reduced by higher physical risk [2]. However, data collected from post-lockdown Wuhan suggested that the negative effect of perceived risk might be temporary. It seemed that people’s perceived risk of COVID-19 did not affect their intentions to travel after lockdown restrictions were lifted in Wuhan. Under a pandemic, the decline in tourism demand is mostly driven by people’s perceived risk of being infected. When people feel control over the disease, the impact brought by COVID-19 may become more limited. In fact, a consumer survey on tourism in China shows that both the airline-seat capacity and the hotel-occupancy rate have gradually recovered in the country since late February. In particular, Chinese people’s confidence in domestic travel increased as much as by 60% in May 2020 [36]. While it is unsure if the global tourism industry will recover at the same speed in the post-pandemic era, it is still encouraging to learn that the effect of COVID-19 perception might be limited once the disease is contained.

### Cultural Influence

Another factor that may possibly explain the difference in travel intention between the two cities is socio-cultural influence. As a paradigm for cross-culture research, Hofstede’s cultural dimensions theory has been frequently employed by scholars [37]. In his work, Hofstede proposed six dimensions of national culture, namely, power distance, uncertainty avoidance, individualism-collectivism, masculinity-femininity, long-short term orientation, and indulgence-restraint [37]. Among these six dimensions, uncertainty avoidance is considered as an appropriate benchmark to compare cultural effects in the field of tourism studies [5]. Previous research reveals that cultures with high uncertainty avoidance, such as Japan, would tend to take on shorter trips with fewer destinations, whereas cultures with a medium level of uncertainty avoidance would demonstrate more risk-accepting behaviours [38]. Based on Hofstede’s ranking of 76 countries on uncertainty avoidance, Japan ranks 11th with an index value of 92 while China is among the least uncertainty avoiding countries with a score of 30 [37]. In light of this, it is possible that Chinese people in general would incline to take more risks and travel even under the COVID-19 pandemic.

### Effect of COVID-19 on Information Processing

This study was conducted in part to verify whether the dual-route information processing theory can be applied to understand how people reach their risk judgments. Analysis results showed that while both routes successfully contributed to COVID-19 perception in Sapporo and post-lockdown Wuhan, neither seemed to function when Wuhan was still in lockdown. This finding raised an interesting question as whether traditional information processing approaches would fail under an extreme public health crisis. Research on the nature of perceived risk demonstrates that people’s risk judgments are often fallible partly due to media biases [39]. In the case of Sapporo, where the total number of confirmed cases was much lower compared to Wuhan in April [11], the majority might not feel affected and rely mostly on media to learn about the disease. On the other hand, people who had personal experience with the disease, such as people in Wuhan during lockdown, might have not estimated their personal risks based on what they heard but what they witnessed. The predictive role of personal experience on risk perception was also confirmed in past research [40]. When daily life was set back to normal in post-lockdown Wuhan, however, people might start to feel distant from the disease again. Consequently the influence of external information might come back into play after the disease was under control.

## Conclusion and Implications

Drawing upon the elaboration likelihood model (ELM), the present study examined how the risk perception of COVID-19, predicted by argument quality and source credibility, could affect people’s travel intentions. The study results confirmed that while perceived risk related to disease would affect people’s travel intentions, its effect might be short-lived or limited. This can be supported by an earlier study on the influence of 2009 H1N1 influenza, in which people’s perceptions of the disease did not directly affect their travel intentions [18]. In addition, this study also examined the validity of the dual-route processing theory under a global pandemic. As hypothesized, self-efficacy successfully predicted people’s information processing. However, the study results suggested that the dual-route information processing model could only go so far in predicting risk perception when the respondents were not directly affected by the event. Since previous dual-route theory research concerning risk perception rarely focused on people who were actually affected by the events [19, 20], further investigation would be needed to verify this finding.

Several practical implications can be found in the present study. Tourism recovery ties in closely with disease containment. In order to stop the disease from spreading, local authorities should devote more efforts in raising people’s awareness of COVID-19. Since self-efficacy can improve people’s processing of COVID-19 information, providing basic knowledge of the disease may help people build a correct understanding of the current pandemic situation. On the other hand, since the negative effect of COVID-19 perception seems to be temporary on people’s travel intentions, hospitality and tourism practitioners may rest assured after the disease is contained as long as they can maintain expected hygiene standards.

Nevertheless, some limitations should be addressed regarding the present study. First, the internal consistency of items measuring COVID-19 perception was relatively low in Wuhan both during and after the lockdown. Given that all items were adapted from the literature [18] and the Cronbach’s alpha value of the same construct in Sapporo was well above 0.8, the measurement should be adequate for the research purpose. Considering that people in Wuhan were the first affected by COVID-19, their perceived risk of the disease might be outside the scope of the survey questions. Thus, a new set of measurements may be needed in future research to assess risk perception in regions that are most affected by the event. Second, while the present study inferred that the dual-route processing model might fail to function in a public health crisis, other possible dependent variables, such as perceived information usefulness, were not measured in the first round of data collection. Future studies should include as many variables as possible to broaden the understanding of the dial-route processing theory under extreme conditions. Lastly, it should be noted that the majority of the respondents in the second Wuhan study were relatively young compared to the other two groups. Since adolescents and young adults had a higher probability of being asymptomatic when infected with COVID-19 [41], they might be less concerned about the disease and thus have higher intentions to travel when restrictions are lifted. A sample with age diversity may be helpful to investigate the relationship between perceived risk and travel intention in the post-pandemic era.
